# A Password Meter without Password Exposure

**DOI:** 10.3390/s21020345

**Published:** 2021-01-06

**Authors:** Pyung Kim, Younho Lee, Youn-Sik Hong, Taekyoung Kwon

**Affiliations:** 1 Advanced Software Research Center, Incheon National University, Incheon 22012, Korea; firimir@gmail.com (P.K.); yshong@inu.ac.kr (Y.-S.H.); 2 ITM Programme, Department of Industrial Engineering, Seoul National University of Science and Technology, Seoul 01811, Korea; 3 Graduate School of Information, Yonsei University, Seoul 03722, Korea

**Keywords:** authentication, privacy, computer security, network security, cryptography

## Abstract

To meet password selection criteria of a server, a user occasionally needs to provide multiple choices of password candidates to an on-line password meter, but such user-chosen candidates tend to be derived from the user’s previous passwords—the meter may have a high chance to acquire information about a user’s passwords employed for various purposes. A third party password metering service may worsen this threat. In this paper, we first explore a new on-line password meter concept that does not necessitate the exposure of user’s passwords for evaluating user-chosen password candidates in the server side. Our basic idea is straightforward; to adapt fully homomorphic encryption (FHE) schemes to build such a system but its performance achievement is greatly challenging. Optimization techniques are necessary for performance achievement in practice. We employ various performance enhancement techniques and implement the NIST (National Institute of Standards and Technology) metering method as seminal work in this field. Our experiment results demonstrate that the running time of the proposed meter is around 60 s in a conventional desktop server, expecting better performance in high-end hardware, with an FHE scheme in HElib library where parameters support at least 80-bit security. We believe the proposed method can be further explored and used for a password metering in case that password secrecy is very important—the user’s password candidates should not be exposed to the meter and also an internal mechanism of password metering should not be disclosed to users and any other third parties.

## 1. Introduction

In password-based authentication, a user must provide a pair of an id and password to a server, in order to gain access. For the purpose, the user posing as a client should enroll to the server with the pair in an early stage. A password is usually a string of up-to-tens of characters, each of which users should type with a keyboard or on a touchscreen. Password-based authentication is still widely used, although it is insecure as warned in many previous research works: users are not good at memorizing longer character strings while advancement of adversaries’ computational power demands longer passwords to expand the password space [1].

**Figure 1:**
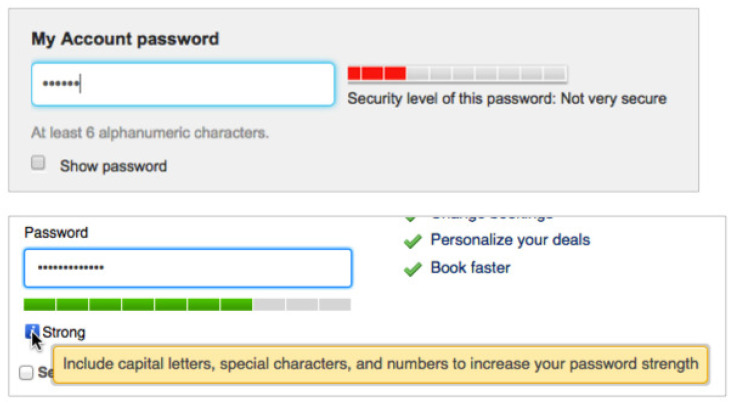
Typical password meters.

Password metering is one of the promising solutions to deal with this problem. A password meter obtains a candidate password from a user who wants to register a password, then evaluates whether a given candidate password supports a sufficient level of security [2,3,4]. Figure 1 shows a conventional password meter. As in the upper part of Figure 1, a password meter normally shows the conditions that a strong password should meet, such as “at least six alphanumeric characters”. It also displays the strength of the candidate password using the length of a bar or colors. It may express the security level of the candidate using appropriate terms such as ‘very weak’, ‘weak’, ‘secure’, ‘very secure’, or etc.

In this way, the user’s security awareness is improved by allowing the password meter user to choose a stronger and more secure password. Therefore, it is very important for the password meter to provide accurate strength feedback to the user, and research on the password meter has focused on improving its accuracy. The criteria for password strength is generally the number of guesses or the time it took to crack the password. In the NIST document [5], the entropy is measured according to the configuration of the password as a standard for recommendation the usage of strong passwords, and the entropy means the range(resolution) of a password that an attacker needs to estimate the password. The most representative methods are studies that generate password candidates and measure the number of guesses using Probabilistic Context-Free Grammar (PCFG) and n-gram Markov chain model [4,6,7,8,9,10,11,12,13,14]. These stochastic approaches analyze frequently used words, patterns, and grammar structures in passwords. Recently, research has been conducted to improve the performance of such password guessing by using machine learning [15,16,17,18,19].

However, few studies have been conducted focusing on the security of the environment in which the password meter runs, not on the accuracy. Studies on estimating the strength of passwords through password guessing suggest that password guessing methods can themselves be used as attack methods. In fact, well-known password cracking tools such as John the Ripper [6] and Hashcat [14] are examples. Research on various password meters ironically provides an effective tool for attackers. If an attacker can analyze the password strength meter that the server uses to prevent the client from generating weak passwords, this has a potential benefit in that the attacker can take a way around it. Information about the dictionary used by many password meters, most easily conceived, is the benefit that an attacker can gain from analyzing password meters. An attacker can increase the performance of an attack tool by simply removing the words included in the dictionary used by the server from his or her dictionary. How to check the repetition pattern and measure the strength according to the length of the password are effective information for an attacker. Therefore, the password meter must be carefully managed and it is also important to keep the password meter up-to-date.

Password meters can be classified into two types: off-line meters and on-line meters. In off-line meters, the metering program is downloaded to the client and runs locally. Thus, the candidate passwords are not exposed to any entity, which provides the secrecy of the candidate passwords. On the other hand, it provides an opportunity for attackers to easily obtain the dictionary used by the meter and to analyze how passwords are measured. It may affect the security of the system. Maintaining up-to-date meters are more difficult task in off-line metering, as clients need to check whether the meter is up-to-date whenever they need to update their passwords. On-line meters are easy to maintain because the important parts of meters are in the server. The dictionary used need not be exposed, which gives less chance to the attackers learn the words in the dictionary. Thus, if sufficient amount of computation power is supported in the server, on-line metering looks preferable.

Unfortunately, there is a crucial problem in on-line metering: the candidate passwords are delivered to the meter, thus they are exposed to the meter. Therefore, if the meter is malicious, users’ security may be breached. For example, suppose you are registering a website run by the government. In this case, if you enter a number of candidate passwords to the on-line password meter of that website. The intelligence agency of your country may crack your other accounts easily with the collected password candidates.

The purpose of this research is to resolve this problem: we build up a password meter such that the meter cannot acquire the information about the clients’ candidate passwords. Nobody except the client who provides the candidate password knows its the security level.

**Figure 2:**
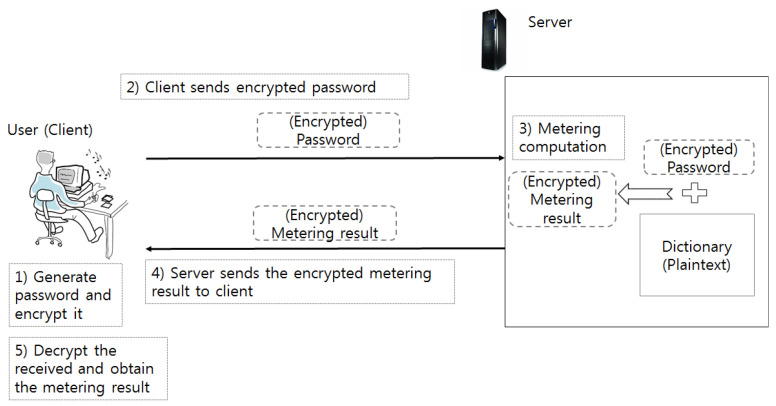
Encrypted password metering scenario.

Figure 2 shows an overview of the proposed password meter. In order to realize the proposed meter, we employ a fully homomorphic encryption (FHE) [20,21,22]. In an FHE scheme, any efficient algorithm can be implemented with the operations that are supported by the FHE scheme. This implementation works as a conventional implementation. However, the difference is that it takes ciphertexts that contain the values that can be inputs of a conventional implementation, and the output is also of an encrypted form, which can be decrypted with the same key as it was used in encrypting the inputs. More specifically, the password meter’s input, strength measurement results, and information such as dictionary used by the password meter must all be protected.

Our new approach’s contributions are as follows:

- We propose a new type of on-line password strength meter that can be secured during the password strength measurement process even if the client does not run the password meter directly. More specifically, the password meter’s input, strength measurement results, and any information such as dictionary used by the password meter are all protected. Therefore, since the proposed method can be run on the server, it can keep the password meter up-to-date and reduce the chances of an attacker to analyze how the password meter works.
- We have achieved an efficient implementation of the entropy-based NIST metering [5] which includes the dictionary membership operation with the encrypted password candidates from a number of different users that use different public keys to encrypt their (candidate) passwords. The experiment demonstrates that a single password metering operation can be completed only with 67 s without exposing the password candidate with a parameter supporting a reasonable level of security. We believe this is the first password meter preserving the password secrecy of the users. Even though there are more advanced metering techniques than the NIST metering, we also believe these can be implemented as our approach when the performance of FHE will be increased in the near future.
- Unfortunately, simply applying an FHE scheme just makes an impractical implementation because of the nature of FHE. Thus, we apply various performance enhancement techniques in order to minimize the required computation cost for metering. Specifically, the dictionary contains more than 50,000 words and the membership operation can be executed even without performing a single recryption operation.
- Our last contribution is to defend against side-channel attacks by ensuring that the password meter always has the same execution time. Password meters that measure strength using password guessing are terminated as soon as the generated guess matches the input password, and the measured strength is correlated with the number of password guesses, that is, execution time. Therefore, if the server can infer at which step of the password meter the match was made by checking the execution time of the password meter, the range of the input password can be greatly reduced. The proposed method prevents such an attack by performing the operation provided by the FHE over the ciphertext according to the same prescribed procedure.

The rest of the paper is organized as follows. Section 2 provides preliminary and related work, which is followed by the motivation in Section 3. Section 4 provides the proposed protocol. Then the performance evaluation is done in the following Section. The final section makes conclusion.

## 2. Preliminary and Related Work

We present the related work in the password-based authentication, including password cracking, modeling, user survey, and metering. Then, we introduce FHE briefly. Finally, we explain the NIST metering [5], which is implemented with a password secrecy-preserving manner in this work.

### 2.1. Recent Literature on Password Cracking and Metering

Password cracking methods can be classified into two types: in on-line cracking, the attacker provides his guessing passwords of a legitimate user through networks. On the other hand, the off-line cracking assumes the attacker has the hashed passwords of legitimate users so the attacker can easily check if his guessing password is correct by matching the hashed result of the guessed password to the (obtained) hashed passwords of the legitimate users. Brute-force attack and dictionary attack are two traditional methods for password cracking. Recently the brute-force attack is not working well because of salting [1]. It is known that the dictionary attack uses a dictionary that contains the words that people select as passwords with high probability. By applying word-mangling rules to extend the dictionary, the dictionary attack can increase its success rate [6,23]. Recently, when using a dictionary collected by password leakage such as the Xato password corpus [24], such an attack has been shown to be more efficient. Habib et al. and Ur et al. [25,26] suggest that a password is generated based on Xato, thereby generating a reasonably strong password.

One of the widely-known questions in this area is if we can calculate how good a password is in terms of security. A possible solution is the NIST password metering method [5]. In this method, the entropy of a given password in calculated in order to measure the security of it. The intention of the method is to calculate the randomness of the password: as more different types of characters can be included, the size of the character set from which each character in the password is increased, which makes the size of the password space greater. Increasing the length of the password leads to a greater password space. Unfortunately, what this approach misses is that passwords are not purely randomly chosen and the distribution of the passwords is biased by the users’ preferences. For instance, they choose easily memorable passwords and they consider their personal information in making their passwords. Some researches such as Weir et al. [27] claim that the NIST method somewhat overestimates the strength of passwords if their sizes are longer than a certain threshold.

Currently, using guessability is a common approach to calculate the strength of a password. It counts the required number of ‘guesses’ for attackers to reach the password. The required number of guesses is defined as the strength of the password. To make this approach meaningful, the attacker should use smart attack methods that can reflect the real-world attackers’ ability well. Therefore, one of the research directions in this field is to pursue a smart attack method. Based on the definition of the guessability, we can easily guess that a good attack method tries more frequently used passwords earlier to find a user’s password because it is more probable that more frequently used passwords are the passwords of users, which the attacker is finding, than less frequently used passwords.

To achieve this purpose, various password cracking methods have been proposed [4,6,7,8,9,10,11,12,13]. They focus on analyzing the structures of the users’ passwords. There are two representative approaches: Probabilistic Context-Free Grammar (PCFG) and n-gram Markov chain [4,7,8,13,14]. Both measures the probabilities that some specific patterns of strings are included in passwords. Thus, they can efficiently calculate the probability that a given candidate password is used by users based on the combination of the probabilities of the occurrences of all the components in the candidate password.

To learn these probabilities, they utilize the list of the exposed passwords to the public such as the RockYou list [28]. There were some other approaches to model the distributions of the user passwords such as Malone and Maher [29] and machine learning method such as Bengio and Courville [30].

If the above cracking methods are combined with the guessability measure, each password’s strength can be measured. Surely, password meters can be built up with the cracking methods and the guessability measure [23,27]. Apart from this approach, there is a work utilizing heuristic rule-based password meter [2]. Komanduri et al. [31] guides users the next character to strengthen their passwords whenever they type a character as a password. Zhang et al. [32] analyzes the relation between the old passwords and the renewed passwords because of password expiration.

There have been some user studies and comparison works in this area. Dell’Amico et al. [12] applies [6,7] on real-world user password sets and shows the success rate of them. De Carné de Carnavalet et al. [3] compares various password meters maintained by commercial companies such as Google, Apple, Microsoft, DropBox [33], and Drupal. With the dictionary that is extended from the well-known cracking tools such as John the Ripper [6] by including the words after mangling and leet transformation, it presents how much portion of the words in the dictionary are regarded as secure passwords in each of the password meters. Kelley et al. [8] analyzes the strength of the passwords made with various composition rules based on the guessability measure. Mazurek et al. [10] analyzes the strengths of the passwords from the people in a university, with a variant of the PCFG model [7]. It analyzes the relation between users’ passwords and their personal information. Egelman et al. [34] conducted research on the effect of password meter in terms of the users’ choice of passwords. Recently, Ur et al. [11] compares the previous password analysis methods such as [4,7], the well-known cracking tools such as John the Ripper [6] and Hashcat [14], and a human-involved proprietary cracking method by a password-cracking expert organization KoreLogic that uses its own dictionary, mangling rules, mask list, and a cracking algorithm based on Markov chain in cracking passwords. The performance analysis in Ur et al. [11] shows that the performance of KoreLogic is the best. The performance of the combination of all four password analysis and cracking methods was comparable to that of KoreLogic. The Password Guessability Service (PGS) [13] is a tool for estimating password strength based on the research in Ur et al. [11] and supports the results of neural networks modeling.

In recent years, with the development of AI technology, research on the use of deep learning as a password guessing technique has been actively conducted. The first password guessing method [15] using deep learning shows that neural networks can be used as a password guessing technique, replacing the PCFG and Markov models. Melicher et al. [15] uses the leaked password list as Recurrent Neural Network(RNN) [35]’s training data, and creates a unit password for each character in the password guessing process. In the case of Hitaj et al. [16], which uses deep learning as described above, a Convolutional Neural Network(CNN) is used as a generator and discriminator of GAN [36] in the process of guessing passwords. From Nam et al. [17,18], RNN and IWGAN were combined to improve performance, and in particular, Nam et al. [18] improved its performance by using a dual discriminator architecture. Nam et al. [19] is a follow-up study of them. It does not simply use the leaked passwords as training data, but selectively uses candidates that are more realistically probable. Apart from that, Pasquini et al. [37,38] studies use the semantic correlation to provide lightweight deep-learning and easy-to-understand feedback to users. Pasquini et al. [37] model the representation of leaked passwords in the space of an instance of Generative Adversarial Networks [39] generator. It performs a conditional password generation using geometric relationships. Pasquini et al. [38] extends this to the character level.

The password meters covered in this subsection focus on measuring password strength more accurately, but the security threats of the running environment, mentioned in Section 1, are not considered. Unfortunately, to the best of the authors’ knowledge, the research on the protection of both the user’s password candidate and the result from the password metering service has not been conducted. We believe this is the first research on this topic. When choosing a new password to log in to a service and receiving feedback on its strength, there is a concern about whether the information of the password candidates is exposed to the outside. In that respect, it makes sense only to pass the password directly encrypted by the user to the password meter, and the password meter which will be explained in the rest of this paper, can cope with the security threats by not leaving any information other than the encrypted password candidate inside the system.

### 2.2. Fully Homomorphic Encryption

The fully-homomorphic encryption (FHE) supports a set of boolean operations over two binary strings that are hidden in the ciphertexts. The supported operations in the set are universal, i.e., any efficient algorithm can be implemented using the operations supported by an FHE scheme. They work with encrypted inputs [20]. The output is also ciphertexts that can be decrypted with the same key as with which the input can be decrypted correctly.

We use HElib (Homomorphic Encryption Library) [22] that implements a variant of Brakerski et al. [21]. HElib’s implementation is explained in [22,40,41]. The following algorithms are supported by the FHE scheme in HElib:

- $\mathrm{Setup}(1^{\lambda })$ takes the security parameter λ and outputs a system parameter params.
- SecretKeyGen(params) generates a secret key sk with params that is an output of running Setup.
- PublicKeyGen(params,sk) takes params and sk and outputs a public key pk that is associated with sk.
- Encrypt(params,pk,m) performs encryption with params, a public key pk, and a plaintext m. m can be one of various forms: from one-bit data to a vector of many bits depending on the way of implementation. It returns a ciphertext c.
- Decrypt(params,sk,c): it decrypts c to the original plaintext m if c is a result of running Encrypt(params,pk,m) where pk is the public key that corresponds to sk.
- Encrypted_XOR(params,pk,$c_{1}$,$c_{2}$): it performs an xor operation with the underlying plaintexts of $c_{1}$ and $c_{2}$. If $c_{1}$ and $c_{2}$ have one-bit plaintexts, it performs a bit-wise xor operation with them. If the underlying plaintexts of $c_{1}$ and $c_{2}$ are represented as vectors, it performs a slot-wise xor operation on each component of $c_{1}$ and $c_{2}$, respectively. One assumption to make this algorithm work is that both $c_{1}$ and $c_{2}$ are encrypted with the same public key. Otherwise it does not return a meaningful output. The result of this operations is a ciphertext *c* that can be decrypted with the secret key with which $c_{1}$ and $c_{2}$ can be decrypted.
- Encrypted_Multiply(params,pk,$c_{1}$,$c_{2}$): It performs the multiplication operation on the underlying plaintexts of $c_{1}$ and $c_{2}$. It could be a slot-wise multiplication if the ciphertexts have vector plaintexts as in the case of Encrypted_XOR. This operation is the same as the encrypted ‘AND’ operation if the underlying plaintexts, or each of their slot values, are just either 0 or 1. If they are defined over a certain ring, this operation also follows the defined multiplication over the ring.
- Recrypt(params,pk,c) can be explained after introducing the ‘noise’ of a ciphertext. In many of FHE schemes, a ciphertext contains a small amount of noise when it is created for security reasons. Whenever we perform some computation with ciphertexts, the resultant ciphertext has more amount of noise. If it is greater than certain amount, the ciphertext cannot be decrypted, so before it reaches the amount, we need to do this operation to reduce the amount of the noise it has. In fact, it creates a new ciphertext of the same plaintext that the parameter ciphertext c has with less noise. However, the resultant ciphertext initially has more noise than a ciphertext that is created by Encrypt().
- Pack(params,pk,$m_{0}$,$m_{1}$, ⋯, $m_{l-1}$): If the plaintext space is a vector space, we can define this operation. This returns an element m in the plaintext space where the components are $m_{0}$, ⋯, $m_{l-1}$. The length *l* and the type of each $m_{i}$s depend on the plaintext space.
- UnPack(params,pk,m) is a reverse of Pack() algorithm. The output is $m_{0}$, ⋯, $m_{l-1}$.
- Shift(params,pk,c,*t*) shifts the position of the plaintext component in each slot to its left by *t* slots. This is defined only when the plaintext space is a vector space. *t* is an integer, and it can be negative. In this case, it means shifting right by |t| slots. We assume that the first *t* slots are set to zero. If it is negative, the last |t| slots are set to zero.
- Rotate(params,pk,c,*t*) returns a new ciphertext where *t*-th slot’s underlying plaintext value in c is in the first slot of the resultant ciphertext. *t* is a multiple of *u* that is defined by the plaintext space. It does not consider what the other slots’ values of the resultant ciphertext are.
- Frobenius(params,pk,c,*t*) produces a new ciphertext where the binary polynomial in each slot of c is exponentiated by $2^{t}$, respectively.

#### A Plaintext Can Be Regarded as a Correct (but Not Secure) Ciphertext That Is Encrypted by Any Public Key

One important property of the HElib’s FHE scheme [21,22] is that a plaintext of a correct form can be treated as a valid ciphertext syntactically so we can suppose that it is a ciphertext generated from any public key. We can perform Encrypted_XOR() or Encrypted_Multiply() with a plaintext and a ciphertext if they were created using the same params. The following is a more detailed description of some algorithms in the FHE scheme in HElib [21,22].

- Setup($1^{\lambda }$) returns params=(R,d,n,q,χ,N), where R: ring description, *d*:degree, *n*: dimension, *q*: odd modulus, χ: noise distribution, and N=N(λ)=$n\dot{p}olylog\left(q\right)$.
- SecretKeyGen(params): Sample t from $\chi^{n}$ randomly. Let $s:=\left(1,t\right)=\left(1,t_{0},t_{1},\;\cdots ,\;t_{n-1}\right)$$\in R_{q}^{n+1}$. Output sk=s.
- PublicKeyGen(params,sk) generates a matrix $B\to R_{q}^{N\times n}$ uniformly at random. A column vector with ‘small’ coefficients $\hat{e}$ is sampled from $\chi^{N}$ at random. Set $\hat{b}:=Bt^{T}+2\hat{e}$. Set $pk:=A:=(\hat{b}||-B)\in R_{q}^{n+1}$. Output pk. (Note that $A\cdot sk^{T}=A\cdot s^{T}=\hat{b}\cdot 1-B\cdot t^{T}=2\hat{e}$).
- Encrypt(params,pk,m): $m\in R_{\in }$. Set a row vector $m^{\prime }:=\left(m,0,\;\cdots ,\;0\right)\in R_{q}^{n+1}$. Sample a column vector with small coefficient $\hat{r}$ from $R_{2}^{N}$ at random and output the ciphertext $c:=\hat{c}:=m^{\prime }+{\hat{r}}^{T}\cdot A\in R_{q}^{n+1}$.
- Decrypt(params,sk,*c*): Calculate $m_{out}:={\left[{\left[<c,sk>\right]}_{q}\right]}_{2}$, where ${\left[\right]}_{q}:R\to \left[-q/2,q/2\right)$ denotes the modular reduction function that reduces a real number *x* into an integer in the range [−q/2,q/2) if *q* is an odd modulus. If q=2, ${\left[\right]}_{q}:=mod\;2$ over real numbers.

If we take a look at Encrypt(), $c=m^{\prime }=\left(m,0,\;\cdots ,\;0\right)$ holds if we assume $\hat{r}={\left(0,\;\cdots ,\;0\right)}^{T}$. Thus, $m^{\prime }$ can be a ciphertext of any public key because c=m holds regardless what A=pk is if $\hat{r}={\left(0,\;\cdots ,\;0\right)}^{T}$. We call this procedure ‘nullified’ encryption because it does not hide the encryption and can be performed without any user’s public key.

### 2.3. NIST Meter

The NIST SP-800-63 document [5] recommends a method that borrows the concept of information entropy in order to represent the strength of a password. We can determine the strength of a password with the method. In the method, the entropy of a given password is calculated then the amount of time to crack the password is estimated. The estimated time can be treated as guessability, the metering result of the password.

In the method, initially, the entropy of the password of which we want to measure the strength is set to zero. whenever a password meets a specific rule respectively, a certain number of bits are added to its entropy. The followings are the rules and their corresponding bit-entropy.

After the entropy of the password is calculated with Table 1, we derive the required crack time with the following formula:

(1) $$\begin{array}{c} crack\_time=2^{entropy-1}\times 0.0001 \end{array}$$

The Formula (1) assumes that the attack can try one password in 0.0001 s. If it changes, Formula (1) changes, too. With the calculated cracking time, we can decide the strength of a given password using Table 2.

## 3. Motivation

Typically, an embedded password meter is activated while people register their passwords into an on-line service. The on-line service accepts their passwords only when the password meter guarantees its strengths above a certain threshold. Since a password metering is included in the password registration procedure, each on-line service maintains its own password policy [11]. Thus, even the same password is assessed to have different strengths depending on which on-line service’s meter is used. Because of that, people need to use so many different passwords for each on-line service, which makes remembering their passwords difficult. To overcome this, we can think of an independent password metering service, where passwords’ strengths can be measured in a unified way.

Unfortunately, this approach causes the problem of revealing the passwords of users in various services to the password meter. Thus, the security of the password meter would be extremely important. Users and services must also trust the password meter.

The motivation of our research lies here. We would like to break the assumption regarding the high-security requirement and the trustiness requirement of the password meter: by making it possible for the password meter to determine a given password’s strength without exactly knowing the password. If it is possible, we can build up this kind of unified password meter more easily: it can contribute to increasing the strength of users’ passwords.

Even in the current situation, such a password meter is very useful in that users tend to provide multiple password candidates to register. Some of them might be used for other services and the users may not want to reveal them to the service that does not need them for authentication.

In light of this, we suppose that the password meter is a passive adversary who follows the honest-but-curious model.

## 4. Proposed Method

In this work, we realize an on-line password meter that supports the NIST password metering with a high level of password privacy, even to the password meter itself. The main technique we use to achieve this is FHE: with the operations supported by FHE, we can implement the NIST meter which takes an encrypted input and produces an encrypted output. However, simple straight-forward implementation of the meter cannot achieve reasonable performance: our experiment found that this requires more than a day to check a password. Therefore, we utilize many optimization techniques to reduce the computation cost. The notations that will be used in this section are given in Table 3.

### 4.1. Overview

**Figure 3:**
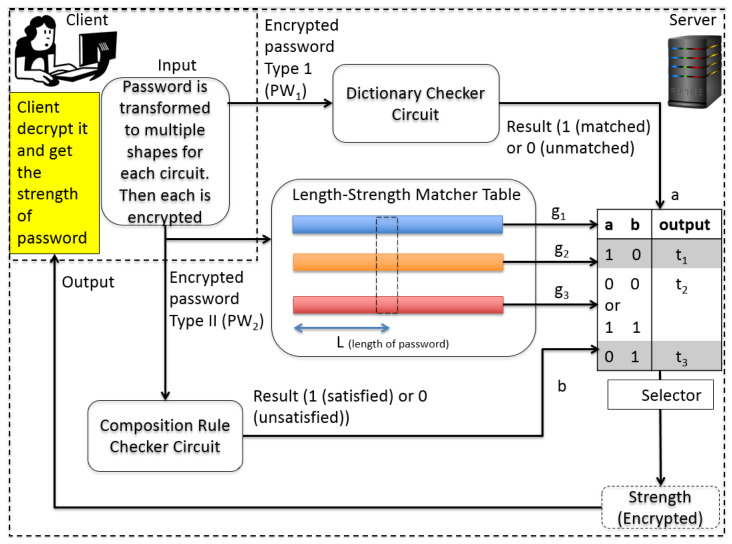
Overview of the proposed meter.

Figure 3 shows an overview of the proposed protocol. At the beginning of the protocol, a client sends the server two encrypted forms of the password she wants to meter. The server performs some computation with the encrypted passwords after receiving the encrypted passwords. Then, it sends the output back to the client. The client can obtain the strength of the password after decrypting the result with its key. We assume that the client encrypts her password correctly as the protocol instructs. She cannot obtain a correct password metering result if she sends an inconsistent password.

The main part of the proposed protocol is the server part. Every component is working with encrypted inputs, and its output is also of an encrypted form. In order to reduce the overhead, the proposed scheme should have as little circuit-depth as possible. The width of the circuit in each component should be minimized because once the depth of the circuit exceeds a certain threshold, a recryption operation begins and the number of recryption will be dependent on the width of the circuit at that circuit level. The main components of the server implementation are three-fold: the dictionary checker (1), the composition rule checker (2), the access of the length-strength table (3). They are independent circuits in terms of the multiplication operation so the total multiplicative depth is not so high.

The result of each component is collected in the final selector circuit (4) to determine the final output. We, fortunately, have found a way to implement the server part without even a single recryption, which takes normally at least a couple of minutes even with a very efficient parameter setting in a conventional desktop environment.

Algorithm 1 shows the pseudo-code description of the server part. Multi-threading is applied in DictionaryChecker(). After that, four independent threads are working before invoking Selector().
**Algorithm 1:** Pseudo-code of the proposed protocol
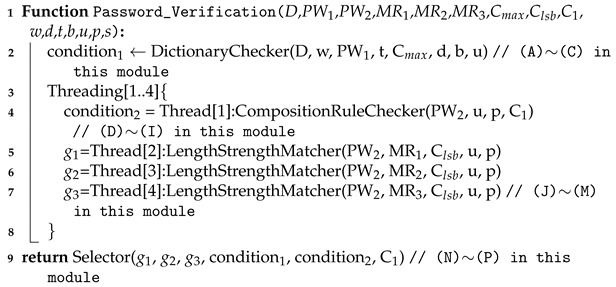


We explain these components and the ways to encode the input password before encryption to make PW${}_{1}$ and PW${}_{2}$ interpret their output in the following subsections.

### 4.2. Dictionary Checker

**Figure 4:**
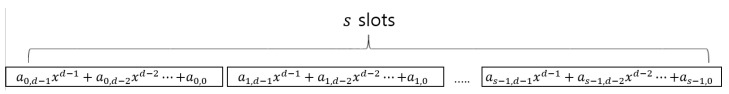
The structure of a ciphertext.

The most time-consuming component is the dictionary checker because more than 50,000 words need to be compared with the input candidate password being metered. One of the challenges in building this component is how to encode words in the dictionary in an efficient way. Basically, a ciphertext contains a number of polynomials of binary coefficients that are defined over a certain polynomial ring. The number of polynomials and their degrees are decided by the parameter used. Figure 4 shows the structure of a ciphertext [40,42]. Every slot contains a binary polynomial of degree *d* (i.e., Every $a_{i,j}\in \left\{0,1\right\}$). There are orders among the slots. We can compute either an addition (i.e., XOR between coefficients in the same position) or a multiplication between the same slots in the two ciphertexts.

Users can obtain the coefficients after decryption. The multiplication is a polynomial multiplication over $GF(2^{d})$ with an irreducible polynomial.

Normally, only the constant terms are used to encrypt a plaintext because multiplication is the same as a bit-wise AND operation in this case. We can embed more bits to other coefficients. However, if so, the multiplication operation is not the same as the bit-wise AND operation thus, it is very difficult to manipulate the underlying bits in the ciphertext.

A naive approach to implement the dictionary checker is to embed the words in the dictionary into multiple ciphertexts and they are compared with the ciphertext that contains the password. In this case, for example, if we use the ASCII encoding to represent a character, because it needs 7-bits to encode a character, in total, 7p slots are required to embed a word into a ciphertext, where *p* is the longest word in the dictionary. Then the number of ciphertexts required to embed all the words in the dictionary is $\left\lceil n/\left(\frac{s}{7p}\right)\right\rceil$. We also embed the password ⌊s/(7p)⌋ times into a single ciphertext. Then, we perform the XOR operations with the ciphertext of passwords and each of the ciphertexts that contain the words in the dictionary. Then, if the password is in the dictionary, one consecutive *p* slots have zero values. This approach has two problems: one is that too many ciphertexts are required because only one bit is used in every slot. Another is that to check whether consecutive *p* slots have zeros we need a lot of multiplication operations, which leads to an intolerably long time to obtain the result.

We have found a solution to overcome these problems. In our approach, one slot contains one word. Thus, only ⌈n/s⌉ ciphertexts are needed to embed all the words in the ciphertexts. We use a secure hash function that takes a string of an arbitrary bit-length and produces a *d* bit output. Every word in the dictionary is hashed and the hashed result is included in the ciphertext. The same thing is done with the password. Thus, PW${}_{1}$ is a ciphertext that is composed of the hashed password in every slot. Now, there is still a question that is unresolved: how can the search operation be performed? We utilize a clever technique. The point is the field inversion operation. After xoring each of the dictionary ciphertexts with the password ciphertext. The slot-wise field inversion operation is performed on each output of the xor operation. In this case, the result is a non-zero element in $GF(2^{d})$ in every slot, except the case where the input slot of the inversion operation has a zero element (this function is called the trace function [40]). By performing the multiplication operation with the input of the inversion and the output of the inversion, we can obtain a ciphertext where every slot has either 0 or 1. The existence of the zero-value slot means it is highly likely that the password is in the dictionary. Then, the result of this multiplication is xored with a ciphertext that contains only one in every slot. As a result of the final operation, the resultant ciphertext contains a slot of one only when there is a match between a hash of the password and a hash of a word in the dictionary. The cost of the inversion operation is inexpensive thanks to the Frobenius map. Of course, we have false-positives. However, we can reduce them significantly by utilizing multiple hash functions. We talk about this issue in the next section. In addition to this, we make the algorithm multi-threaded so that it can work faster if there are multiple computation units.

The Algorithm 2 describes the DictionaryChecker() function. The sub-functions used in the DictionaryChecker() are also described in Algorithm 3. Trace(X[], d) computes the multiplicative inverse of the element X[] and performs the multiplication with the original element. If the original element is zero, the result is also zero. Note that ResultXOR(R${}_{1}$[],w,t) executes xoring the first w elements in R${}_{1}$[] in parallel with t threads, and m-Multiply(X[],d) performs the encrypted multiplication on the first d elements in X[]. We omit the detailed description due to the page limit of the initial submission.
**Algorithm 2:** Pseudo-code of Dictionary Checker
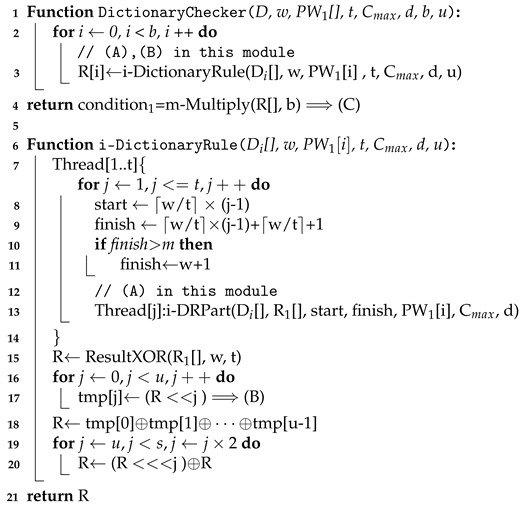
**Algorithm 3:** Pseudo-code of i-DRPart(), WordMatcher(), and Trace() function.
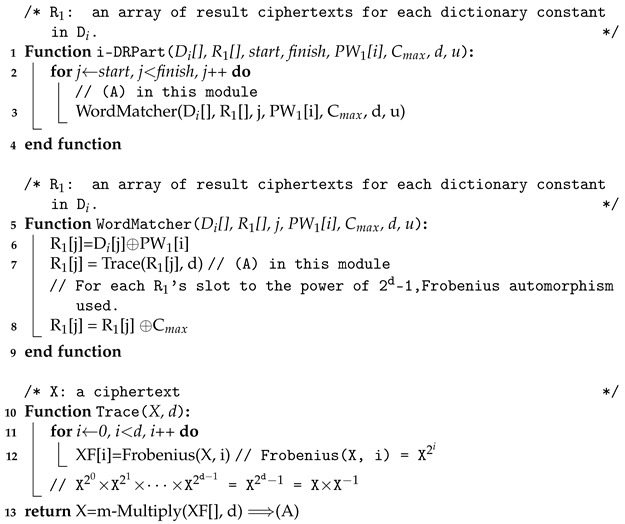


**Figure 5:**
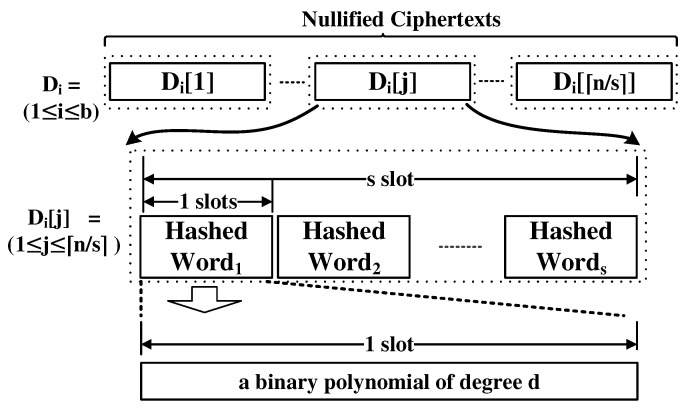
Dictionary encoding.

Figure 5 shows the dictionary encoding. each D${}_{i}$[j] contains the nullified ciphertexts of the hashes of *s* dictionary words.

**Figure 6:**
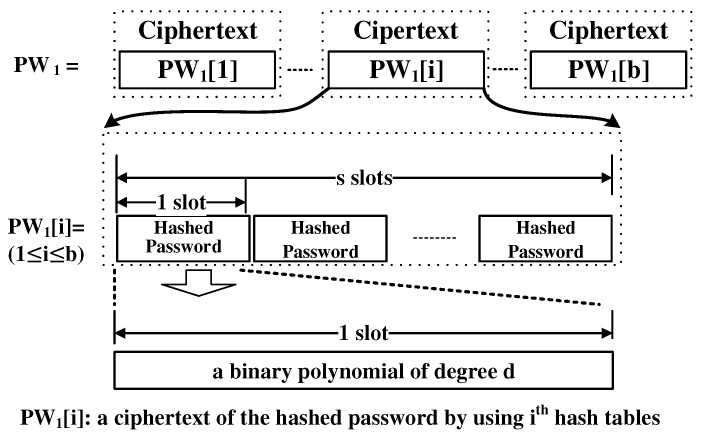
PW${}_{1}$ encoding.

Figure 6 shows the encoding of PW${}_{1}$. PW${}_{1}$ is composed of b ciphertexts, where each PW${}_{1}$ [i] (1 ≤i≤ b) has the result of hashing the password with the i-th hash function in each slot. Each slot represents a binary polynomial of d-degree. Note that the ciphertexts in PW${}_{1}$ are encrypted by the user’s public key.

### 4.3. Composition Rule Checker

**Figure 7:**
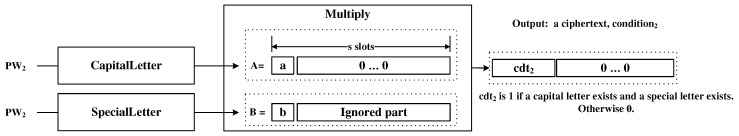
Composition Rule Checker.

This checker determines if a given (encrypted) password includes both a capital letter and a special letter. It is composed of the circuits: CapitalLetter and SpecialLetter, as shown in Figure 7. Each circuit just outputs either 0 or 1 in the first slot of the result. By multiplying them, the result can be obtained.

**Figure 8:**
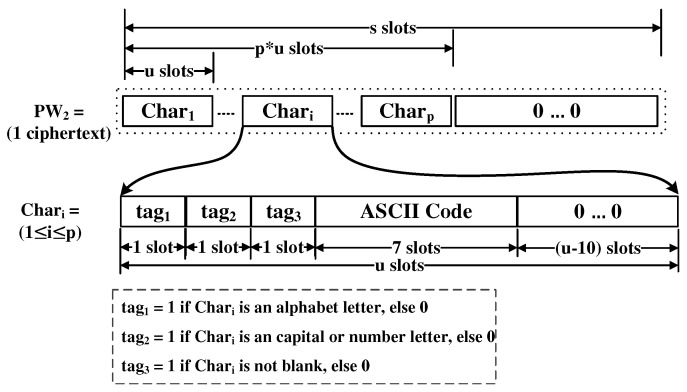
Encoding the characters in password to PW${}_{2}$.

In order to perform it efficiently in the server, the user encodes the password with the following manner. u slots are used to encode a character in the password. Let us call each of these u-slots ${\mathrm{Char}}_{i}$, where i refers to the relative position in the ciphertext that begins with 1. u is greater than 10 and the ciphertext should be able to contain at least up slots. Each tag${}_{i}$ is of 1-slot. The details of the encoding is given in Figure 8. The blank means the character of ASCII code 0.

A detailed description of the composition rule checker is given in Algorithm 4.

It is composed of CapitalLetter and SpecialLetter circuits. CapitalLetter just checks if there is a character where both tag${}_{1}$ and tag${}_{2}$ are set to 1. Since the shift operation is very cheap if the number of slots shifted is a multiple of u, we can efficiently construct it. SpecialLetter checks if there is a u-slot unit where tag${}_{1}$ and tag${}_{2}$ are zeros and tag${}_{3}$ is one.
**Algorithm 4:** Pseudo-code of CompositionRuleChecker().
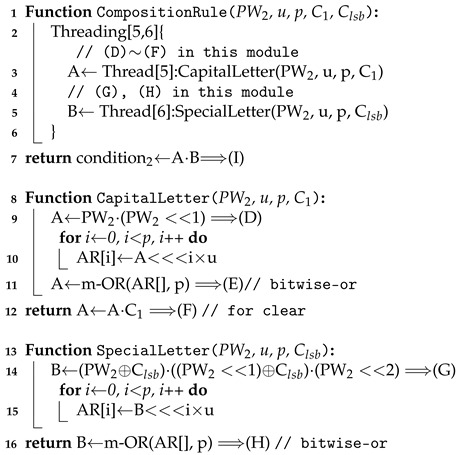


### 4.4. Length-Strength Matcher Table

The main task of this circuit is to calculate the strength of a given password based on its length. However, depending on the results of the composition rule checker and the dictionary checker, the strength becomes different even if we fix the length. There are three cases: the dictionary checker circuit outputs 1 and the composition rule checker circuit outputs 0 (no entropy increasing), the dictionary checker circuit outputs 0 and the composition rule checker circuit outputs 1 (12-bit entropy increasing), and otherwise (6-bit entropy increasing). For each case, we build up a table that maps the length of the password to the corresponding strength of it, respectively. Then, we obtain the results of accessing all three tables and they are sent to the selector.

**Figure 9:**
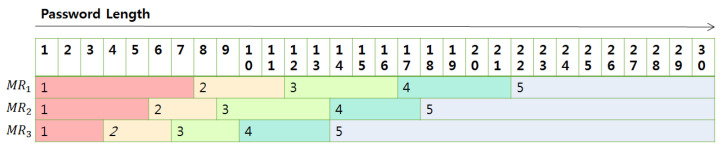
The content of length-strength matching tables; The red color is dangerous and the stronger the password goes to the right.

Now, let us explain how to construct tables. In each case in the above, if the length of the password is determined, we can calculate the total entropy. Then, with the entropy, we can calculate the crack time with which we can grade the password strength using Table 2. We put this strength value in the table where the index corresponds to the length of the password used to calculate it. The following Figure 9 shows the content of the tables.

**Figure 10:**
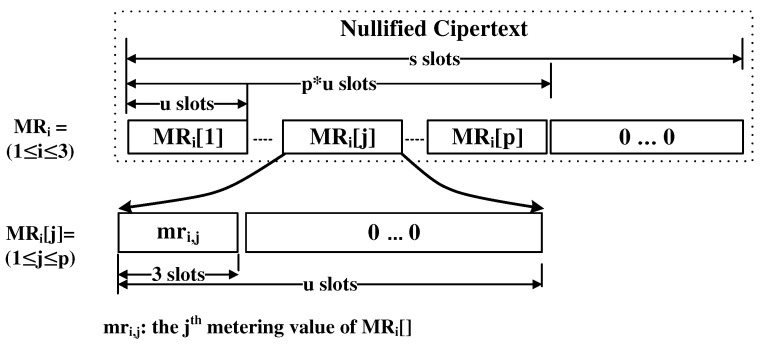
Length–strength matcher table.

Actually, we build up tables such that each table is a ciphertext where indexes are represented using the relative order among *u*-slot-unit positions in the ciphertext, and the corresponding strengths are encoded in the first three slots in the u-slot unit. The structures of the tables MR${}_{1}$, MR${}_{2}$, and MR${}_{3}$ are as follows in Figure 10. To come up with the PW${}_{2}$, i-th value in the table is included in the i-th u-slot unit in the corresponding ciphertext.

Since we do not know which value will be used as the final result. We access all three tables to obtain the corresponding value of an encrypted form.

Now let’s talk about how to access each table. In PW${}_{2}$, tag${}_{3}$ indicates if the current character is a blank or not, which means that every tag${}_{3}$ is zero after the last character of the password. We use this to make the first three slots in the u-slot unit, which is mapped to the last password character, are set to one and the rest of the slots are set to zeros. If it is multiplied by the MR${}_{i}$, only the values that are mapped to the length of the password are alive and the others are set to zeros. We copy this value to the first three slots in every u-slot unit in the resultant ciphertext using the rotation operation. This procedure is formally described in the Algorithm 5. The results g${}_{1}$, g${}_{2}$, and g${}_{3}$ which are the execution result of the Algorithm 5 with MR${}_{1}$, MR${}_{2}$, and MR${}_{3}$, respectively, are sent to the selector.
**Algorithm 5:** Pseudo-code of LengthStrengthMatcher()
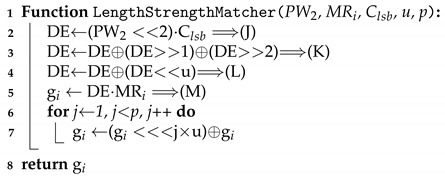


### 4.5. Selector

We can easily construct the selector with the outputs of the dictionary checker and the composition rule checker so that only the intended output among g${}_{1}$, g${}_{2}$, and g${}_{3}$ remains in the output of the selector in the Algorithm 6.
**Algorithm 6:** Pseudo-code of Selector()
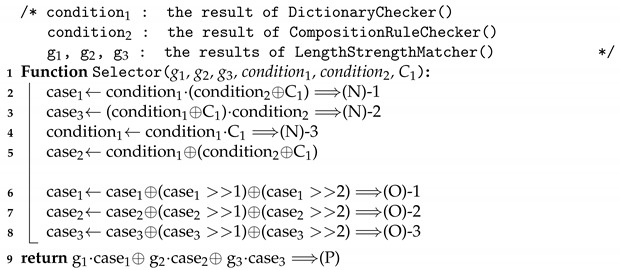


## 5. Performance Evaluation

This section analyzes the proposed method in terms of computational cost and communication cost. In the first subsection, we analyze the proposed method in detail. We first provide the number of operations required in the proposed circuit. Then, we provide the depth of the proposed circuit and the width of the circuit in each depth in order to predict the performance of the proposed circuit in various parameters easily. In the second subsection, we show the evaluation result by running the proposed scheme in a conventional desktop environment. We provide the size of the compressed ciphertexts that we made in the experiments.

### 5.1. Theoretical Analysis

#### 5.1.1. Number of Unit Operations in Components

We first analyze the number of unit operations in each circuit in Table 4. Because we assume the multi-threading is possible, each component is divided into multi-threading part and non-multi-threading part, and analyzes the number of operations in each part separately, in terms of the various parameters. The most dominant operation is the multiplication operation in the multi-threading part of the dictionary checker, which requires $O(\frac{b\cdot d\cdot n}{s\cdot t})$.

#### 5.1.2. Circuit Depth and Width Analysis

Because the recryption operation is quite heavy and slow, we need to analyze how many recryptions may happen on various parameters. To achieve this, we have to analyze the proposed method’s required multiplicative depth and the width of the circuit in each multiplicative circuit-level because a recryption operation is needed whenever a certain multiplicative depth is reached in computation from the last recryption, and the width at that depth determines the number of recryptions operations necessary.

**Figure 11:**
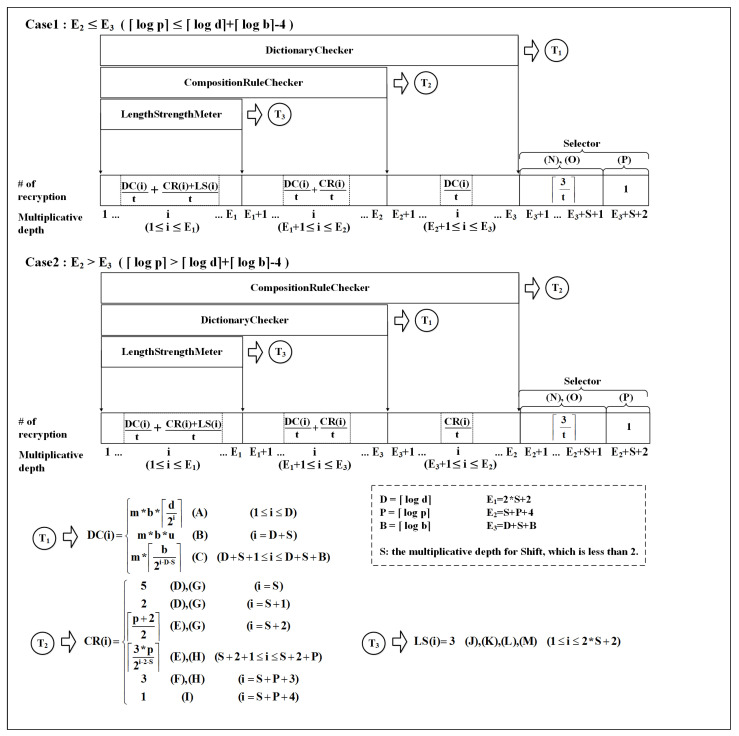
Multiplicative depth analysis.

With the analysis of them, practitioners can easily estimate the required recryptions on the various parameters that are not covered in this paper. Figure 11 shows the analysis result. The main part is the Gantt chart where the number of required recryption is given in each multiplicative depth if the recryption operation is occurring at that depth. The three components of the circuits run in parallel in terms of increasing circuit depth. Thus, the number of recryptions in a given circuit depth is determined by the sum of those in each component at that depth.

The result is divided into two cases based on the condition on the size of p, d, and b. The total multiplicative depth is very close to the greater value in ⌈log d ⌉+⌈log b⌉+ 4and8 +⌈log p⌉ because S < 2.

(A)∼(P) in Figure 11 are mapped to (A)∼(P) in Algorithms 1–6. They refer to the lines that affect the multiplicative depth and width analysis.

### 5.2. Experimental Analysis

We have performed an experiment on the execution of the proposed method in a conventional desktop environment. The machine has Intel(R) Xeon(R) ES-1650 v3 @ hexa-core processor From Intel, Santa Clara, CA, USA. where twelve threads can be executed in parallel. It also has 64GB RAM. Our implementation was executed on Ubuntu 16.04LTS. We utilized HElib 1.2 [22] with the parameters shown in Table 5, which is written in C++14 and uses the NTL 11.3.2 mathematical library. We set p=32 because the longest word in the dictionary is 32. In the word list file of [6], *n* = 52,801 dictionary *D* was constructed with only English words.

#### 5.2.1. Unit Operation Time

**Figure 12:**
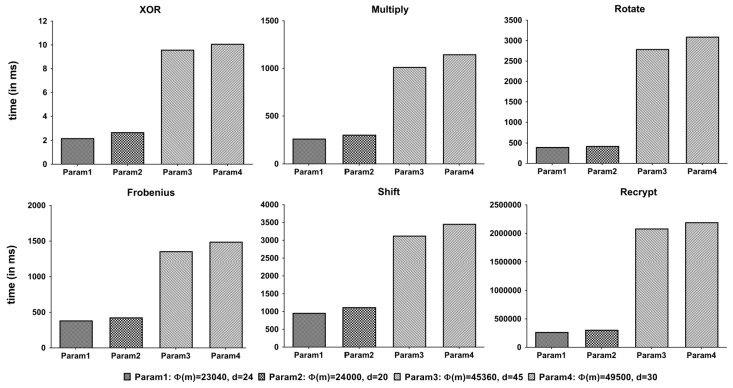
Execution times of unit operations.

We measured the execution time of each operation by 1000 times. The average result is shown in Figure 12. The execution time depends on the size of the parameter m shown in Table 2.

**Figure 13:**
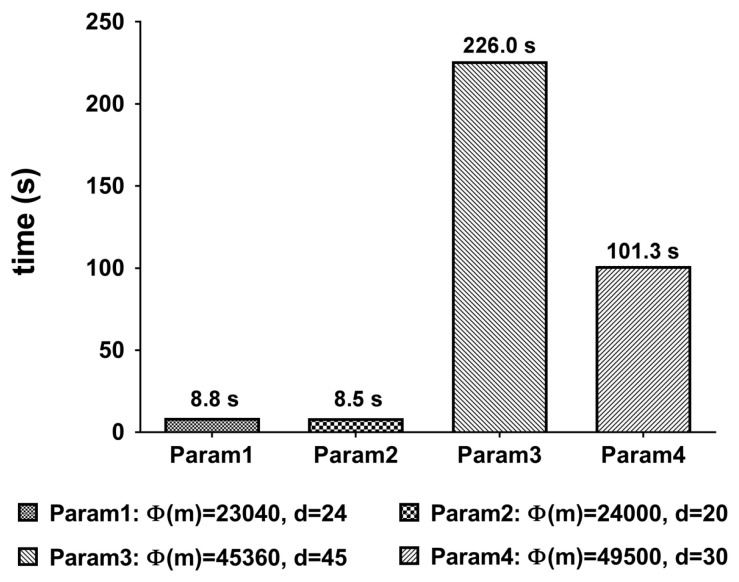
Execution time of the trace function.

Figure 13 shows the execution time of the trace function on various parameters. Since we use this function to check if a given encrypted password is contained in the dictionary, it is used a lot of times. From the figure, we can see that it also looks highly dependent on the size of the ring (m).

From Figure 12 and Figure 13, only the first two parameters are competitive in terms of execution time.

#### 5.2.2. False Positive Ratio in Dictionary Checking

**Figure 14:**
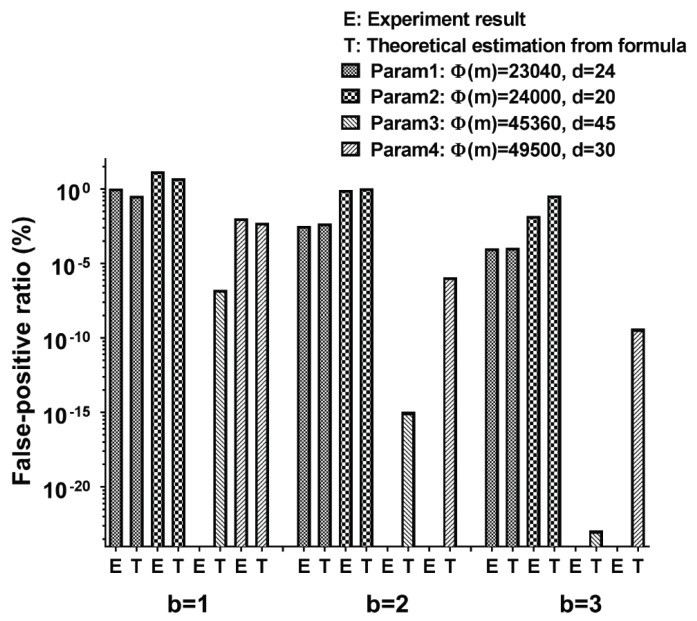
False positive ratio in dictionary checking on various b and parameters.

Since the proposed approach utilizes a bloom filter [43], we have to analyze the false-positive ratio in the dictionary checking. We have performed experiments with the parameters that are introduced at the beginning of this section. In each experiment, we generate 1 million random passwords whose lengths are in the range between 20 and 30 characters (inclusive), where each character can be typed. By regenerating the password when the generated password is in the dictionary, we could avoid any word in the dictionary is included in the generated random passwords. Then, we run the hash function with them and check if their hash values are included in the hash values of the words in the dictionary. We could count the number of random passwords whose hash values are the same as any word in the dictionary, and it is the number of times hash collisions occur. In other words, when performing a dictionary-check, it corresponds to a false-positive for determining that there is a random password that does not exist in the dictionary. Figure 14 shows the false-positive ratio value from both experiments and the conventional formula to derive the false-positive ratio in the bloom filter. The formula derived from [43] is $\left(FP\right)\approx {\left(1-e^{-kD/N}\right)}^{k}$ where *D* is fixed to 58201 (i.e., # of words in the dictionary), k=b, and $N=2^{d}$. We can see that the cases where the estimated false-positive rate is below $10^{-5}$ % produce zero collision in the corresponding experiments. We also could check that the practical parameter (Param1 with b=1) just produces 1% of the false-positive rate.

#### 5.2.3. Total Execution Time

Table 6 shows the experiment result regarding the total execution time. The composition rule checker and the length strength meter are working in parallel. So, only one of them whose execution time is longer than the other affects the total execution time. Actually, the dictionary checker is an independent circuit but it needs all the threads supported by the environment. Thus, the other components cannot be executed until the dictionary checker ends its execution.

In the proposed method, the process that requires the highest computational overhead is DictionaryChecker, which checks the dictionary *D* containing *n* = 58,201 words, and the overall performance is significantly different in Table 6 by the execution time of DictionaryChecker. As can be seen in Figure 13, the Trace function occupies the highest proportion in DictionaryChecker. Since the trace function calculates the inverse of the polynomial that composes the slots of the ciphertext, the higher the degree d of Table 2, the higher the computational overhead. In addition, since b is the number of hashes that encode the dictionary *D*, the number of ciphertexts that DictionaryChecker should perform increases in proportion to b. From the Table 6 we can see that Param1 and Param2 with b=1 support reasonably fast execution time.

#### 5.2.4. Key Size for FHE

We measured the size of the public key pk and secret key sk of FHE for each parameter setting given in Table 2, and the result is shown in Table 7. pk is required to encrypt the password candidate to be passed to the password meter and to perform operations over the encrypted data provided by the FHE in the password meter. In the case of sk, it is only needed when decrypting the final result, and this key is not shared with the password meter.

#### 5.2.5. Ciphertext Size

We measured the size of a single ciphertext in order to estimate the communication cost of the proposed protocol. The result is given in Table 8. The client gives two ciphertexts, PW${}_{1}$ and PW${}_{2}$, to the server and the server gives the client a single ciphertext that contains the metering result. From Table 8, we need the resource to transmit several MB data through the network. Note that the individual length of the password used in the proposed method does not affect the execution time and size of the ciphertexts. In the case of PW${}_{1}$, since it is encoding using a hash function, it is irrelevant to the password length. In the case of PW${}_{2}$, as shown in Figure 8, the maximum length of the password that can be available is p=s/u calculated as *s* and u given in Table 2. All the processes of the proposed method are performed in the form of the encrypted password, and the password meter cannot be executed by distinguishing the length of the password used. Therefore, it can be seen that only the maximum length p affects the execution of Algorithms 4 and 5. We limited the p to 32, which can be considered long enough in the experiment.

## 6. Conclusions

In this paper, we have proposed a new type of on-line password metering scheme where the meter is very difficult to obtain the information about the password being measured. The proposed method takes an encrypted password as input, measures its strength, and returns the result in the encrypted form. In this process, the password meter performs the operation provided by FHE over the ciphertext according to the prescribed procedure. Thus, this gives us a high level of privacy to the passwords being tested. The fact that the password check is performed according to the same predefined procedure for all encrypted inputs can prevent side-channel attacks of the server, which can be made by measuring the execution time of the password meter.

Table 9 is a comparison of the existing password meters with the proposed method for several factors that affect security. The proposed method can utilize a lot of resources on the server-side and is easy to maintain the password meter as up-to-date as possible. The proposed password meter does not disclose any information because the input password, the measured strength, and the dictionary used are all encrypted. This is in contrast to the existing methods where the candidate password whose strength is being measured and the dictionary should be of a plaintext form, which helps the hackers to crack users’ passwords a lot if they are exposed.

Additionally, we utilized FHE with clever implementation techniques such as some smart ways of encoding the passwords and Bloom Filter to make the proposed scheme of practical performance. This approach enhances password security because the users do not have to reveal their passwords in metering.

We also believe that the FHE application techniques used in this work can be used for many other applications requiring privacy, such as machine learning [44] and Internet of Things [45] fields.

## Figures and Tables

**Table 1:** Entropy assigning rules in NIST meter [5] (*L*: the length of password).

Condition		Entropy
Length	1	Add 4 bits
	2∼8	Add 1.7+2.3L bits
	9∼20	Add 8.1+1.5L bits
	21∼	Add 18.1+L bits
Existence of Non-alphanumeric characters and capital letters		Add 6 bits
A dictionary of more than 50,000 words does not contain the password		Add 6 bits

**Table 2:** Metering result calculation from the crack time (in s).

Crack Time	Grade of Password Strength
∼$10^{2}$	very-weak
∼$10^{4}$	weak
∼$10^{6}$	so-so
∼$10^{8}$	good
>$10^{8}$	great

**Table 3:** Notation.

Notation	Description
*n*	The number of words in the dictionary used
*s*	The number of plaintext slots that can be contained in a ciphertext
*p*	The maximum length of a word and a password in characters
*L*	The number of the characters in the password being checked
*w*	The number of the nullified ciphertexts of the dictionary used, ⌈n/s⌉
PW${}_{i}$	*i*-th representation of a password of a plaintext form
*d*	The plaintext space in a slot is defined as $GF(2^{d})$.
*t*	The number of threads with which the password meter can run to execute the meter.
*u*	The minimum number of the slots that can be shifted without heavy noise increasing.
<<<,>>>	a rotation operation without heavy noise increasing
<<,>>	a shift opertion that requires heavy noise increasing
·	the encrypted multiplication operation
⊕	the encrypted exclusive or operation
*S*	the multiplicative depth level of a shift operation
*b*	the number of the hash tables being used
$MR_{i}$	*i*-th length-strength matcher table
*m*	The degree of the cyclotomic polynomial of binary coefficiens.
$D_{i}\left[\left\lceil n/s\right\rceil \times j+l\right]$	The *l*-th ciphertext of thread #j which has the hashes of the *s* words of from the (⌈n/s⌉×j+l−1)×s+1-th to the (⌈n/s⌉×j+l)×s-th order in the dictionary. The *i*-th hash function is used.
C${}_{max}$	a constant with all slot set by 1.
C${}_{lsb}$	a constant with the first of each *k* slot set by 1.
C${}_{1}$	a constant with the first slot set by 1.
*D*	$\{D_{1}$[], $D_{2}$[], ⋯, $D_{b}\left[\right]\}$: the set of hashed dictionaries.
Thread[j]:A()	the function A() is invoked by the thread *j*, which is an independent execution unit.

**Table 4:** Analysis on the number of unit operations.

**Dictionary Checker**				
Non-multithreading part		Multithreading part		
Operation	Number of operations	Thread no.	Operation	Number of operations
XOR	$b\cdot \left(u-1+\lceil \log\frac{s}{u}\rceil \right)$	1∼t	XOR	$b\cdot \left(2\cdot \left\lceil \frac{n}{s}\right\rceil /t+\left\lceil \frac{n}{s}-1\right\rceil /t\right)$
Multiply	b−1	i-DRPart	Multiply	$b\cdot \left(d-1\right)\cdot \left\lceil \frac{n}{s}\right\rceil /t$
Rotate	$b\cdot \left(\lceil \log\frac{s}{u}\rceil \right)$		Rotate	0
Frobenius	0		Frobenius	$b\cdot \left(d-1\right)\cdot \left\lceil \frac{n}{s}\right\rceil /t$
Shift	b·(u−1)		Shift	0
**Composition Rule Checker**				
Non-multithreading part		Multithreading part		
Operation	Number of operations	Thread no.	Operation	Number of operations
XOR	0	1	XOR	2·(p−1)
Multiply	1	CapitalLetter	Multiply	p+1
Rotate	0		Rotate	p−1
Frobenius	0		Frobenius	0
Shift	0		Shift	1
		2	XOR	2p
		SpecialLetter	Multiply	p+1
			Rotate	p−1
			Frobenius	0
			Shift	2
**Length Strength Matcher**				
Non-multithreading part		Multithreading part		
Operation	Number of operations	Thread no.	Operation	Number of operations
XOR	0	1∼ b	XOR	p+2
Multiply	0		Multiply	2
Rotate	0		Rotate	p−1
Frobenius	0		Frobenius	0
Shift	0		Shift	3
**Selector**				
Non-multithreading part		Multithreading part		
Operation	Number of operations	Thread no.	Operation	Number of operations
XOR	12	−	XOR	0
Multiply	6		Multiply	0
Rotate	0		Rotate	0
Frobenius	0		Frobenius	0
Shift	6		Shift	0

**Table 5:** Parameters for experiment.

	Param1	Param2	Param3	Param4
Cyclotomic ring (m)	28,679 =7×17×241	31,775 =$\;5^{2}\;\times \;31\;\times \;41$	46,063 =73×631	49,981 =151×331
Lattice dimension (ϕ(m))	23,040	24,000	45,360	49,500
Plaintext space (=$GF(2^{d})$)	$GF(2^{24})$	$GF(2^{20})$	$GF(2^{45})$	$GF(2^{30})$
Number of Slots (=*s*)	980	1200	1008	1650
Security Level	93	93	86	94
Maximum multiplicative depth to reach the first recryption	22	24	24	24
*u*	10	30	14	11

**Table 6:** Total execution time (in s).

b	Parameter in HElib	Dictionary Checker	Working in Parallel		Selector	Execution Time
			Composition Rule Checker	Length Strength Meter		
1	Param1	53	7	11	3	67
	Param2	50	8	6	3	61
	Param3	1154	25	19	10	1189
	Param4	348	34	30	12	394
2	Param1	108	7	8	3	119
	Param2	101	8	6	3	112
	Param3	2318	25	19	10	2353
	Param4	705	34	30	9	748
3	Param1	162	7	9	3	175
	Param2	153	7	6	3	175
	Param3	3450	25	18	9	3484
	Param4	1055	34	30	9	1098

**Table 7:** A single key size for fully homomorphic encryption (FHE) (in MB).

	Param1	Param2	Param3	Param4
pk	22.149	21.969	86.516	94.414
sk	27.422	27.102	106.593	116.320

**Table 8:** Size of a single (compressed) ciphertext.

	Param1	Param2	Param3	Param4
size (in MB)	3.9	4.3	17	19

**Table 9:** Security Properties of the proposed method in contrast to current meters.

	The Proposed Method	Current Meters
Protection of password candidates to be entered	O	X
Protection of metering results	O	X
Protection of dictionary D, used in the meter	O	Only if meter uses hased dictionaries or nothing.
Maintaining up-to-date	O	Only the on-line meters.

O: protected, X: not protected.
